# Peptidome profiling for the immunological stratification in sepsis: a proof of concept study

**DOI:** 10.1038/s41598-022-15792-5

**Published:** 2022-07-06

**Authors:** Martín Ledesma, María Florencia Todero, Lautaro Maceira, Mónica Prieto, Carlos Vay, Marcelo Galas, Beatriz López, Noemí Yokobori, Bárbara Rearte

**Affiliations:** 1 Laboratorio de Bacteriología, Departamento de Bioquímica Clínica, Hospital de Clínicas “José de San Martín”, Facultad de Farmacia y Bioquímica, UBA, Av. Córdoba 2351, C1120 CABA, Argentina; 2 Instituto de Medicina Experimental (IMEX) - CONICET - Academia Nacional de Medicina, Pacheco de Melo 3081, C1425AUM CABA, Argentina; 3 Servicio de Bacteriología Especial. Instituto Nacional de Enfermedades Infecciosas (INEI), ANLIS “Dr. C. G. Malbrán”, Av. Vélez Sarsfield 563, C1282AFF CABA, Argentina; 4 Special Program of AMR, Communicable Diseases and Environmental Determinants of Health Department, Pan-American Health Organization, 525 23rd St NW, Washington, DC 20037 USA; 5 Departamento de Bacteriología. INEI, ANLIS “Dr. C. G. Malbrán”, Av. Vélez Sarsfield 563, C1282AFF CABA, Argentina; 6 Servicio de Micobacterias INEI, ANLIS “Dr. C. G. Malbrán”, Av. Vélez Sarsfield 563, C1282AFF CABA, Argentina; 7 Consejo Nacional de Investigaciones Científicas Y Técnicas (CONICET), Godoy Cruz 2290, C1425FQB CABA, Argentina

**Keywords:** Inflammation, Experimental models of disease, Diagnostic markers

## Abstract

Sepsis has been called *the graveyard of pharmaceutical companies* due to the numerous failed clinical trials. The lack of tools to monitor the immunological status in sepsis constrains the development of therapies. Here, we evaluated a test based on whole plasma peptidome acquired by MALDI-TOF-mass spectrometer and machine-learning algorithms to discriminate two lipopolysaccharide-(LPS) induced murine models emulating the pro- and anti-inflammatory/immunosuppression environments that can be found during sepsis. The LPS group was inoculated with a single high dose of LPS and the IS group was subjected to increasing doses of LPS, to induce proinflammatory and anti-inflammatory/immunosuppression profiles respectively. The LPS group showed leukopenia and higher levels of cytokines and tissue damage markers, and the IS group showed neutrophilia, lymphopenia and decreased humoral response. Principal component analysis of the plasma peptidomes formed discrete clusters that mostly coincided with the experimental groups. In addition, machine-learning algorithms discriminated the different experimental groups with a sensitivity of 95.7% and specificity of 90.9%. Data reveal the potential of plasma fingerprints analysis by MALDI-TOF-mass spectrometry as a simple, speedy and readily transferrable method for sepsis patient stratification that would contribute to therapeutic decision-making based on their immunological status.

## Introduction

Sepsis constitutes one the major causes of death in intensive care units^1,2^ with an incidence of 31.5 million cases and 7–9 million deaths annually^3^. The impact of sepsis in developing regions such as Latin America is elusive due to the limited number of prevalence studies, but the high incidence rate of infectious diseases and the inequities in the access to health care indicates that sepsis is an alarming health problem in the region^4–7^.

The redefinition by the Sepsis-3 consensus highlights the need to integrate the information on the etiological agent and the host response status and strongly emphasizes that the dysregulated host response as a key component of sepsis^8^.

From the immunological point of view, sepsis is characterized by an unbalanced response in which both hyper-inflammatory and anti-inflammatory/immunosuppressive responses occur concurrently contributing in different degrees to the sepsis immunopathology in each patient^9–12^. Sepsis has earned the epithet of “the graveyard of pharmaceutical companies” due to the numerous failed clinical trials and this could be related to an inappropriate timing for the application of immunomodulatory interventions^11^. The lack of tools to monitor the host immune status constrains the evaluation of novel immunomodulatory therapeutic approaches. Despite the efforts put in biomarker search in the past decades, only a handful of molecules, such as procalcitonin, proved to be useful in the clinical setting under specific conditions, and the fact that efficient biomarkers not necessarily correspond to key mediators adds further complexity^13^. Moreover, none of them determines the quickly changing immunological status of the host^9–11,14–19^.

With the advent of artificial intelligence algorithms including machine learning (ML)^20^, top-down analysis of unlabeled biological samples is gaining ground. This is the case of matrix-assisted light desorption ionization-time of flight-mass spectrometry (MALDI-TOF–MS), a powerful tool that is currently spreading in the clinical setting for microbiological biotyping and in biomedical research for the study of biological samples in several diseases including sepsis^21,22^.

Taking into account that around 50% of all sepsis cases are caused by Gram-negative bacteria^23,24^, we^25–31^ and other authors^32,33^ demonstrated that both inflammatory and anti-inflammatory/immunosuppression processes that occur during sepsis can be emulated by the administration of lipopolysaccharides (LPS) in murine models. These models proved to be useful because they represent in a discrete manner two immunological states that are believed to underlie simultaneously the complex immunopathogenesis of sepsis.

Herein, we hypothesized that the proinflammatory and anti-inflammatory/immunosuppression environments can be discriminated through the analysis of plasma peptidome profiles generated by MALDI-TOF–MS. Thus, in this study, we developed and evaluated the performance of predictive models based on ML algorithms to discriminate the pro- and anti-inflammatory/immunosuppression states in two LPS-induced in murine models.

## Results

### Simulation of pro- and anti-inflammatory states

The proinflammatory state was induced with a high dose of LPS (LPS group), whereas the anti- inflammatory/immunosuppression state (IS group) was reached with a scheme of increasing doses of LPS (Supplementary. Fig. S1). In order to characterize the two models, immunological, inflammatory and tissue damage markers were studied in the two experimental groups as well as in the control group (CTL group). In accordance with our previous results, the LPS group was characterized by a marked leukopenia (Supplementary Fig. S2a), high plasma levels of pro-inflammatory cytokines such as TNF-α but also of the anti-inflammatory cytokines IL-10 and TGF-β (Supplementary. Fig. S3a, b). Tissue enzymes indicative of damage were also elevated (Supplementary Fig. S4a–c).

On the other hand, the IS group showed a leukocytosis due to increased numbers of circulating granulocytes, particularly of polymorphonuclear neutrophils (Supplementary Fig. S2b)^25^, along with high plasma levels of TGF- β and low levels of pro-inflammatory cytokines (Supplementary Fig. S3c). No signs of tissue damage were observed in this group (Supplementary Fig. S4d–f). As previously described^28,30,31^, IS mice had a profound immunological impairment, accompanied by a marked lymphopenia and an increased number of neutrophils in the spleen. In addition, splenic myeloid CD11b cells expressed higher levels of the inhibitory receptor PDL-1 (Supplementary Fig. S5a, b). Moreover, the IS group showed significantly lower antibody titers upon immunization with SRBCs (Supplementary Fig. S5c). Collectively, these results indicate that the pro- and anti-inflammatory/immunosuppression states were emulated in our murine models.

### MALDI-TOF MS data analysis

**Figure 1:**
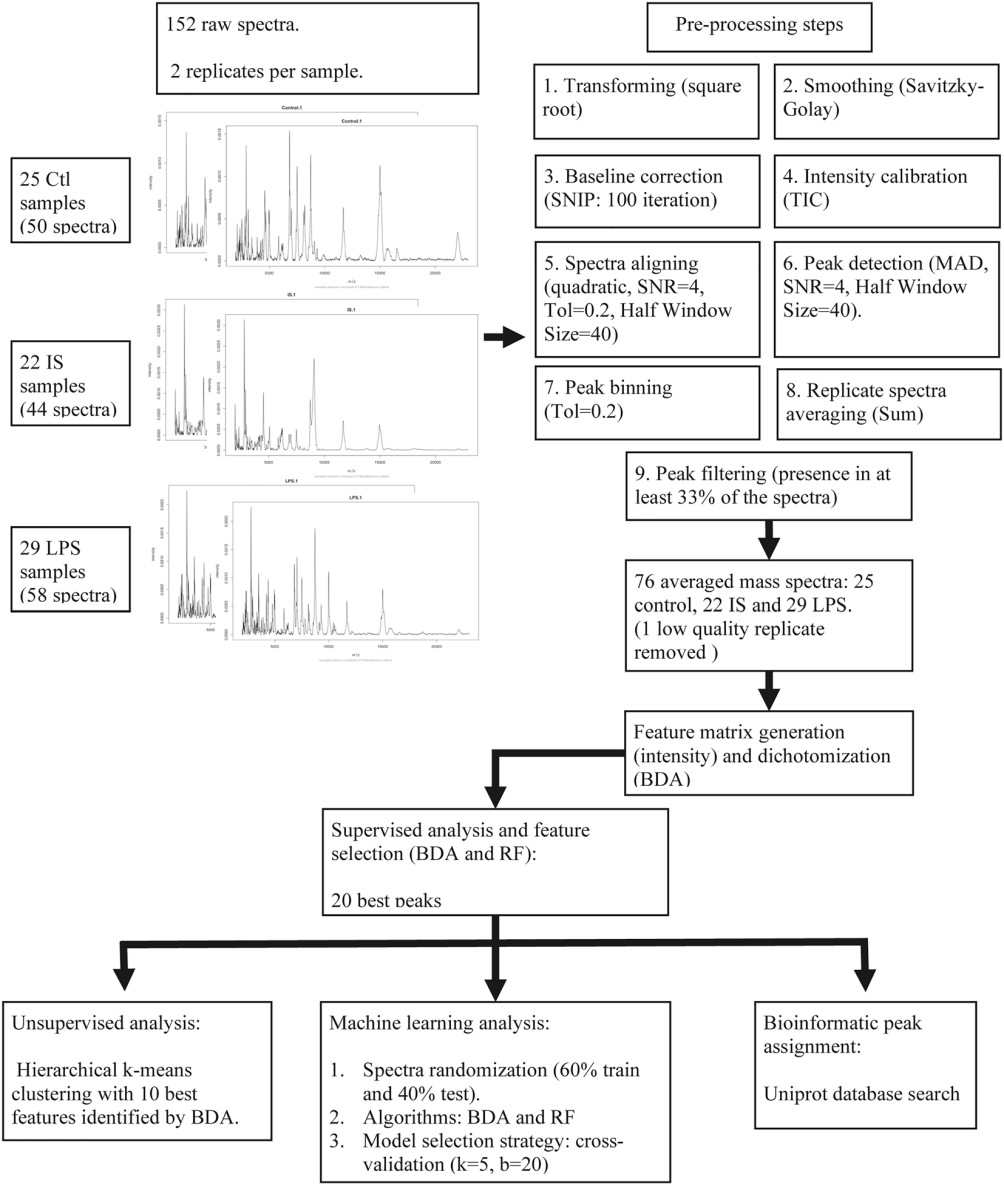
MALDI-TOF- MS data analysis pipeline. CTL, control group; IS, immunosuppression/anti-inflammatory group; LPS, pro-inflammatory group; BDA, binary discriminant analysis; RF, random forest.

**Figure 2:**
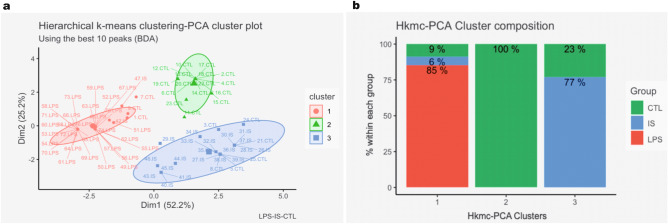
Unsupervised statistical analysis. (**a**) Hierarchical k-means clustering (Hkmc)-Principal Component Analysis (PCA) cluster plot using the top ten peaks selected by the binary discriminant analysis (BDA) algorithm. Labels contain the mice ID and the experimental groups. PC1 (Dim1, x-axis) and 2 (Dim2, y-axis) are depicted. Spectra were clustered into three groups using the Hkmc algorithm, which are represented with three different colors. 95% confidence ellipses were added around cluster means, assuming a multivariate normal distribution. (**b**) Hkmc-PCA cluster composition. The green color represents the CTL mice group, the blue represents the IS group, and the red color represents the LPS group.

Plasma samples of the CTL, IS and LPS groups were analyzed by MALDI-TOF MS in the 2000 – 20,000 Da range. Representative spectra of each experimental group are shown in Supplementary Fig. S6 and the analysis pipeline is detailed in Fig. 1. A statistical analysis set was implemented to test if MALDI-TOF MS data were useful to discriminate the three experimental groups. First, through a supervised classification algorithm we looked for peaks that best differentiated the CTL, LPS, and IS groups. The 20 best discriminant peaks identified by binary discriminant analysis (BDA) and random forest (RF) algorithms are shown in the Supplementary Fig. S7a and b respectively, which partially overlapped. Of the 20 best peaks selected by the BDA algorithm, the 10 most discriminant peaks were chosen for an unsupervised statistical analysis to plot the hierarchical k-means clustering-PCA clusters (Fig. 2a). Clusters appeared as three non-superimposed groups, and the first two principal components explained 76.6% of the variation with good intra cluster homogeneity. Specifically, cluster 2 achieved 100% (16/16) of homogeneity for spectra corresponding to CTL plasma and 64% (16/25) of CTL spectra fell in this cluster, while for cluster 1 an 85% (29/34) homogeneity was achieved for spectra corresponding to the LPS group and 100% (29/29) of LPS spectra fell in this cluster (Fig. 2b). Lastly, homogeneity of cluster 3 was 77% (20/26) mostly represented by spectra of the IS group, with 91% of the IS spectra (20/22) included in this cluster. This result shows that the immunosuppression/anti-inflammatory and pro-inflammatory states have distinctive plasma peptidome signatures that allow their discrimination.

### Machine learning analysis

ML algorithms were used to develop a predictive model based on the plasma fingerprints, using a training data set (14 CTL, 13 IS and 15 LPS samples) challenged with a test data set (11 CTL, 9 IS and 14 LPS samples; Fig. 1). To find the most refined model, a dimensionality reduction was performed through the selection of different numbers of distinctive peaks in the training set to train the algorithms, and the performances on the test set were computed for each condition. Specifically, the top 5, 10, 15, and 20 discriminant peaks for BDA and RF algorithms were evaluated by cross-validation (CV; 5 folds and 20 repetitions) and percentages of accuracy, sensitivity, specificity, negative predictive value (NPV) and positive predictive value (PPV) in the test data set were obtained (Table 1).

**Table 1:** Performance of the classification models with the top 5, 10, 15, and 20 peaks.

Peaks	Algorithm	A (%)	S (%)	E (%)	PPV (%)	NPV (%)
5	BDA	90.6	95.7	80	90.9	89.8
10	BDA	85.6	92	72.3	87.4	81.1
15	BDA	87.6	93.9	74.5	88.5	85.4
20	BDA	90.1	94.3	81.4	91.4	87.3
5	RF	88.2	95.7	72.7	88	88
10	RF	91.2	91.3	90.9	95.5	83.3
15	RF	88.2	91.3	81.8	91.3	81.8
20	RF	94.1	95.7	90.9	95.7	90.9

A, accuracy; S, sensitivity; E, specificity; PPV, positive predictive value; NPV, negative predictive value.

Between models, RF using the best 20 peaks showed the most consistent values, with sensitivity and specificity values above 90%. The BDA model trained with the best five peaks showed the best performance in terms of detection of positive cases, with a sensitivity above 95% (Table 1); this strategy constitutes the simplest method in terms of both the number of required peaks and computational demands. These results display the potential of plasma peptidome fingerprints to develop predictive models.

## Discussion

Sepsis constitutes a highly heterogeneous syndrome of complex pathophysiology. The underlying immunological alterations that include pro-inflammatory, anti-inflammatory and immunosuppressive components^11^ are reflected in septic plasma protein composition and varies over time according to the disease severity^34^.

In the last decades, several circulating markers with recognized functions in sepsis were identified. However, none of these markers showed the specificity and sensitivity required for its clinical use^16,19^. Furthermore, methods that rely on immunolabeling such as ELISA or flow cytometry are highly specific and sensitive for the detection of low abundance markers, but require costly equipment and consumables, as well as specific technical knowhow. In addition, these techniques are time consuming, limiting their clinical usefulness for early decision-making.

Regarding biomarker discovery, proteomics proved to be an important source of information. However, in the classical approaches the plasma samples are subjected to depletion of abundant proteins^22^ and fractionation to identify individual biomarkers^34,35^ which requires more analysis time, specific equipment and higher costs. These constraints led us to explore a top-down strategy that could overcome these difficulties. Thus, having in mind that we aimed to design a readily transferrable method, our approach was meant to be as simple as possible and unlabeled whole plasma were directly read by MALDI-TOF–MS.

Our results showed that the performance of the test with whole plasma and standard acquisition settings used in routine microbiological biotyping were excellent in terms of discriminatory power. First, an ensemble between supervised and unsupervised algorithms allowed the overall discrimination of plasma samples corresponding to the three experimental groups. These results showed that each state comprises a distinctive MALDI-TOF–MS pattern. A recent study reported a top-down plasma analysis showing that differences between samples from healthy donors and cancer patients could be appreciated without the depletion of large abundant proteins by setting a cut-off at 30KDa^36^, supporting our results.

Furthermore, the performance of our ML algorithms challenged with the test dataset, was able to identify basal, pro-inflammatory and anti-inflammatory/immunosuppressive groups in a very satisfactory way, exceeding 90% of accuracy in some of the tested conditions. The cross validation strategy used herein allows a fine-tuning of the models according to particular needs. Thus, depending on the clinical and epidemiological settings, algorithms with higher sensitivity and lower computational demand like our 5-peaks BDA model could be preferred over those with a good overall accuracy.

The peaks found in the 2-20KDa range mostly correspond to small peptides. Plasma peptidome has been proposed as an interesting source of information for diagnostic purposes^36,37^. These peptides are presumably fragments of larger proteins derived from digestion by proteases or non-enzymatic degradation^38^ but the lack of comprehensive digestome/degradome databases is still a limitation to their study^39^. Nevertheless, recent reports demonstrate the importance of plasma peptidome as a useful tool for clinical monitoring in septic shock^40,41^. Although the identification of individual peaks is beyond the objectives of this study, it would be interesting to evaluate them in the future with specific methods with higher resolving power.

A major limitation of this study is related to the animal models chosen. Although LPS-induced inflammation/immunosuppression models are widely used, they do not represent the dynamic physiological changes that occur in sepsis^42^. Endotoxin challenge promotes a faster and transient release of pro/anti-inflammatory mediators compared to the septic processes. Notwithstanding the limitations**,** these models allowed us to define two discrete immunological states that actually occur concurrently during a septic process. For this reason, we considered this was the most appropriate approach to test the potential of MALDI-TOF–MS analysis in the proof-of concept phase. We are currently validating our results in a poly-microbial model of sepsis as cecal ligation and puncture model (CLP), with very encouraging results. Considering the complexity of human sepsis, in terms of organ systems involved, etiological agent/s, presence of co-morbidities, etc., it would be interesting to challenge our approach in several preclinical models in addition to CLP, following the recommendations of the "Minimum Quality Threshold in Preclinical Sepsis Studies’’(MQTiPSS), : An International Expert Consensus Initiative for Improvement of Animal Modelling in Sepsis” guidelines^43–45^ to improve the chances of translation to the clinic.

Finally, our approach has numerous advantages. MALDI-TOF–MS is a simple and speedy method, a critical feature considering the dynamism of sepsis, which is spreading in the clinical setting even in resource-constrained regions. In addition, we used an accessible sample as plasma, without complex pre-processing steps. Finally, we performed our ML analysis in the open source software R that also guarantees the transferability of the bioinformatics tool. In conclusion, our results indicate that plasma peptidome analysis by MALDI-TOF–MS has the potential to be a powerful tool for sepsis patient stratification and therapeutic decision making based on their immunological state.

## Materials and methods

### Animals

Female and male BALB/c mice (8–12 weeks old) were provided by the IMEX-CONICET-Academia Nacional de Medicina, Buenos Aires, Argentina. Animals were maintained under a 12 h light–dark cycle at 22 ± 2 °C and were fed with standard diet and water ad libitum. Animals were bred and housed in accordance with the NIH Guide and Use of Laboratory Animals^46^. Experimental designs were conducted according to the 3 R’s guidelines and approved by the Institutional Animal Care and Use Committee (IACUC) of Institute of Experimental Medicine (IMEX)-CONICET- Academia Nacional de Medicina (Protocol code 71/2019, date of approval 12/27/2019). The authors complied with the ARRIVE guidelines.

### Murine models

All the inoculation and sample collection schemes are depicted in Supplementary Figure S1.

Briefly, for the inflammatory state model (LPS group), BALB/c mice were inoculated with a lethal dose 50 (LD50) of LPS of *Escherichia coli* O111: B4 (100 μg/ mouse; Sigma-Aldrich, St Louis, MO, USA) intraperitoneally (i. p.)^25^. Plasma samples were obtained at 1.5 h and 6 h after the injection. In the anti-inflammatory/immunosuppression state model (IS group), BALB/c mice were inoculated daily with LPS for 10 consecutive days. The inoculation scheme consisted of increasing doses starting from 5 µg/mouse i. p. for the first 4 days, followed by 50 µg/mouse i. p. for 3 days, and 100 µg/mouse i. p. for the last 3 days^30^. Plasma samples were obtained 24 h after the last LPS dose. A third group of mice inoculated with vehicle (saline solution) served as control (CTL group). The plasma in this group was collected at the same time points indicated in the experimental groups. Peripheral blood was collected by submandibular bleeding method in order to maximize the quality of the sample. Part of the heparinized samples were used for blood cell count and the remaining volume was centrifuged twice (400×g, 10 min at 4ºC) and plasma were stored at − 20 °C until analysis.

### Immunological, hematological and biochemical parameters

Peripheral blood leukocytes were counted in a Coulter hematology analyzer (Diatron Abacus Junior Vet, Budapest, Hungary) at 1.5 h and 24 h post LPS for the LPS and the IS groups respectively. Proinflammatory (TNF-α; IL-12p70; IFN-γ; IL-6) and anti-inflammatory cytokines (IL-10, TGF-β) were determined in plasma by ELISA at the indicated time points (OptEIA set; BD Biosciences, San Diego, CA, USA) according to the manufacturer’s instructions. The tissue damage markers creatinine (Cre), alanine aminotransferase (ALT) and aspartate transaminase (AST) were determined in plasma, at 6 and 24 h in the LPS and IS groups respectively, using a kit from BioTecnica (Varginha, Minas Gerais, Brazil) and the MINDRAY BS-200E auto-analyzer according to the manufacturer’s instructions. For the leukocytes and plasma cytokines determinations, the number of animals (n) used per group for each analysis was of n = 6 to 7 mice; for the biochemical parameters, an n = 5 to 7 mice per group were used.

For flow cytometry analysis, 24 h after the last LPS dose, a splenocyte suspension was obtained and the cells were immunolabeled as described previously^30,31^. The following cell types were evaluated: CD4 (FITC-anti-CD4, clone RM4-5) and CD8 (PECy5-anti-CD8, clone 53-6.7) T-lymphocytes, B-lymphocytes (FITC-anti-CD45R-B220, clone RA3-6B2), polymorphonuclear neutrophils (FITC-anti-CD11b, clone M1/70 and PE-anti-Ly6G, clone 1A8). The expression levels of PDL-1 (PE-anti-PDL-1, clone MIH5) on the CD11b gate of myeloid lineage cells were also evaluated. Labeled monoclonal antibodies were obtained from Invitrogen and BD Pharmingen™. Cells were acquired in a Becton Dickinson FACScan flow cytometer using Cell Quest software (Becton Dickinson, San Jose, CA, USA). An n = 6 to 8 mice per group was used for each analysis.

Primary antibody response to sheep red blood cells (SRBC) was evaluated by immunizing animals 24 h after the last dose of LPS (5 × 10^8^ SRBC /mouse, 0.1 ml i. p.). The anti-SRBC antibody titer was evaluated in serum through a hemagglutination assay 7 days after immunization as described previously^30,31^. An n = 8 mice per group was used in these assays.

### Acquisition of MALDI-TOF–MS spectra

Plasma samples were analyzed with the MALDI Biotyper System (Bruker Daltonik GmbH, Bremen, Germany). Total number of plasma samples analyzed per group were n = 29 for the LPS group, n = 22 for the IS group and n = 25 for the CTL group. Plasma samples were analyzed with the standard method for microbial biotyping. Briefly, 1 μl of plasma was loaded onto each spot in duplicate and 1 μl of the matrix (alpha-cyano-4-hydroxycinnamic acid matrix in 50% acetonitrile and 2.5% trifluoroacetic acid) was added to each dried spot.

Continuous mass spectra were obtained with a Microflex LT/SH MALDI-TOF mass spectrometer using the flexControl software version 3.4.135.0. Acquisition conditions were ionization mode: LD + , acquisition method: MBT_FC.par, acquisition mode: qsim, tof Mode: linear, acquisition Operator Mode: linear, and digitizerType: Bruker BD0G5, within a mass range of 2000–20,000 Da. Spectra were obtained in the manual mode, using 60% of laser intensity with 40 laser repetition in each shot and reaching between 400–500 spectra by acquisition. Internal calibration was performed every day following manufacturer’s instructions (bacterial test standard; Bruker Daltonik GmbH).

### MALDI-TOF–MS data pre-processing

We generated a dataset containing 152 mass spectra from plasma samples corresponding to the three mice groups in duplicate. Mass spectra were read as fid/aqus files with MALDIquantForeign (v0.10)^47^ and they were processed using MALDIquant (v1.16.2)^48^ R package. Briefly, spectra were square root-transformed, smoothed using the Savitzky-Golay algorithm, and were baseline-corrected applying the SNIP process across 100 iterations.

The peaks were detected by a function that estimates the noise of mass spectrometry data by calculating the median absolute deviation (MAD). The signal-to-noise-ratio (SNR) was set up in 4, with a half Window Size of 40 and a tolerance of 0.2 for peak binning. Duplicates were averaged except from one replicate corresponding to the CTL group that was removed due to low quality and 76 averaged spectra were subject to further analysis. Peaks that occurred in less than 33% of the spectra were removed. MALDI-TOF–MS data were transformed by a categorization of the peak intensity, performed with the Binda R package^49^ which compares the intensity value with the peak group average, returning 1 when it was equal or higher and 0 when it was lower. The analysis workflow is depicted in the Fig. 1.

### Statistical analysis

#### Immunological, hematological and biochemical parameters

Graph Pad Prism 6 software (GraphPad Software, San Diego, CA) was used for statistical analysis and plotting. The number of mice or biological replicates (n) analyzed in each experiment were the replicates considered for statistical analysis. The n and the repetitions of each experiment are indicated the figure legends. Values are expressed as the mean ± standard error of the mean (SEM) of n samples. No outliers were removed. The assumption test to determine the Gaussian distribution was performed by the Kolmogorov and Smirnov method. For parameters with a Gaussian distribution, the differences between two experimental groups were assessed by unpaired Student’s t test, and for multiple group comparisons, as the differences in peripheral blood leukocytes, were analyzed using a one-way ANOVA followed by a Tukey’s multiple comparison test. For parameters with a non-Gaussian distribution, comparisons between two experimental groups were performed using Mann–Whitney U test. All statistical tests were interpreted in a two-tailed fashion and a P < 0.05 was considered statistically significant.

#### MALDI-TOF–MS data

Statistical analysis and plotting were performed using Rstudio. Programmed peaks selection was performed in the entire dataset seeking for biomarkers of each class representing the different experimental groups by the Binary Discriminant Analysis (BDA) algorithm, which outputs the t.score (Class means vs. Pooled mean) of each peak. The sign of the t.score indicates the presence (positive t.score) or absence (negative t.score) of that peak in each group. A significance level of 95% was achieved if the modulus of the t.score was equal or higher than 2.5. The best-extracted features were then used to perform a hierarchical k-means clustering-principal component analysis (PCA) with the factoextra R package^50^. The binary distance was used to measure dissimilarity between observations by the ward.D2 agglomeration method with a k of 4. In addition, spectra were analyzed with the random forest (RF) classifier to test another classification model based on a different underlying criterion.

Afterwards, two ML methods were applied in the dataset to train both BDA and RF classifiers in R^51^. Initially, the dataset was randomly partitioned into a training set (60% of plasma samples) and a test set (40% of plasma samples). Programmed feature (peaks) selection was performed with the respective algorithms in the training set seeking for discriminant peaks corresponding to each experimental group. The extracted features were then used to train several ML models. Accuracy, sensitivity, and specificity were used to evaluate the performances of all resulting models in the test set by a cross-validation strategy.

## Supplementary Information

Supplementary Information.

## Data Availability

The MS datasets generated for this study as well as the analysis pipelines can be found in GitHub [https://github.com/MarManLed/SepsisData].
